# Allowing for mandatory covariates in boosting estimation of sparse high-dimensional survival models

**DOI:** 10.1186/1471-2105-9-14

**Published:** 2008-01-10

**Authors:** Harald Binder, Martin Schumacher

**Affiliations:** 1 Freiburg Center for Data Analysis and Modeling, University of Freiburg, Eckerstr. 1, 79104 Freiburg, Germany; 2 Institute of Medical Biometry and Medical Informatics, University Medical Center Freiburg, Stefan-Meier-Str. 26, 79104 Freiburg, Germany

## Abstract

**Background:**

When predictive survival models are built from high-dimensional data, there are often additional covariates, such as clinical scores, that by all means have to be included into the final model. While there are several techniques for the fitting of sparse high-dimensional survival models by penalized parameter estimation, none allows for explicit consideration of such mandatory covariates.

**Results:**

We introduce a new boosting algorithm for censored time-to-event data that shares the favorable properties of existing approaches, i.e., it results in sparse models with good prediction performance, but uses an offset-based update mechanism. The latter allows for tailored penalization of the covariates under consideration. Specifically, unpenalized mandatory covariates can be introduced. Microarray survival data from patients with diffuse large B-cell lymphoma, in combination with the recent, bootstrap-based prediction error curve technique, is used to illustrate the advantages of the new procedure.

**Conclusion:**

It is demonstrated that it can be highly beneficial in terms of prediction performance to use an estimation procedure that incorporates mandatory covariates into high-dimensional survival models. The new approach also allows to answer the question whether improved predictions are obtained by including microarray features in addition to classical clinical criteria.

## Background

For models built from high-dimensional data, e.g. arising from microarray technology, often survival time is the response of interest. What is wanted then, is a risk prediction model that predicts individual survival probabilities based on the covariates available. Because of the typically large number of covariates, techniques have been developed that result in sparse models, i.e., models where only a small number of covariates is used. In modern approaches, such as boosting [1] and the Lasso-like path algorithms [2], it is avoided to discard covariates before model fitting, and parameter estimation and selection of covariates is performed simultaneously. This is implemented by (explicitly or implicitly) putting a penalty on the model parameters for estimation. The structure of this penalty is chosen such that most of the estimated parameters will be equal to zero, i.e., the value of the corresponding covariates does not influence predictions obtained from the fitted model.

Often there are clinical covariates, such as a prognostic index, available in addition to microarray features. The former could be incorporated into the model just like an additional microarray feature, but due to the large number of microarray features compared to the typically small number of clinical covariates there is the danger, that the clinical covariates might be dominated, even when they carry important information. Therefore mandatory inclusion for such covariates is needed. When it is also of interest whether use of microarray features can improve over models based solely on the clinical covariates, i.e., the latter are not only included for increasing prediction performance, the parameters of the clinical covariates have to be estimated unpenalized. Only then the resulting model can be fully compared to models based only on clinical covariates, where typically unpenalized estimates are used.

To our knowledge, existing techniques for estimating sparse high-dimensional survival models do not naturally allow for unpenalized mandatory covariates. In contrast, for the generalized linear model class there is a recent approach that fits this need [3]. We therefore extend this one to survival models. As will be shown, this new approach is closely related to the existing high-dimensional survival modeling techniques when no mandatory covariates are present. Therefore, we first review some of the latter, before developing the extension.

Given observations (*t*_*i*_, *d*_*i*_, *x*_*i*_), *i *= 1, ..., *n*, where *t*_*i *_is the observed time to the event of interest for individual *i*, *δ*_*i *_takes the value 1 if an event occurred at that time and 0 if the observation has been censored, and *x*_*i *_= (*x*_*i*1_, ..., *x*_*ip*_)*' *is a vector of covariates obtained at time zero, many approaches for high-dimensional survival data are based on the Cox proportional hazards model for the hazard

(1) *λ*(*t*|*x*_*i*_) = *λ*_0_(*t*)exp(*F*(*x*_*i*_;*β*)),

where *λ*_0_(*t*) is the baseline hazard and *F *(*x*; *β*) is a function of the covariates, depending on a parameter vector *β*. When a linear predictor of the form *F*(*x*; *β*) = *x'β *is used, each element of the parameter vector *β *= (*β*_1_, ..., *β*_*p*_)' specifies the influence of a single covariate. For estimation, the baseline hazard *λ*_0_(*t*) is left unspecified and an estimate $\hat{\beta }$ is obtained by maximizing the partial log-likelihood

(2) $$l(\beta )=\sum_{i=1}^{n}\delta_{i}\left(F(x_{i};\beta )-\log\left(\sum_{j=1}^{n}I(t_{j}\leq t_{i})\exp(F(x_{j};\beta ))\right)\right),$$

where *I*() is an indicator function taking value 1 if its argument is true, i.e., if individual *j *is still under risk just before time *t*_*i*_, and value 0 otherwise.

When the number of covariates is large, maximization of (2) can no longer be carried out by standard techniques. In Lasso-like approaches (using a linear predictor) [2,4] a penalty term *λ*∑_*j*_|*β*_*j*_| is added to the partial log-likelihood (2). The resulting penalized partial log-likelihood then is maximized by quadratic programming techniques or by the more efficient path algorithms [2]. The penalty parameter *λ *can be determined e.g. by cross-validation. Due to penalizing the absolute value, many elements of the resulting estimate $\hat{\beta }$ will be equal to zero, i.e., the solution will be sparse, larger values of *λ *leading to more sparseness. Lasso-like approaches have in addition been developed for additive risk models [5] and accelerated failure time models [6].

An alternative approach for fitting of sparse high-dimensional models is provided by gradient boosting techniques [1,7]. The underlying principle is that of stepwise optimization of a function *F*(*x*; *β*) in function space by minimizing a loss function. For fitting a Cox model, the negative partial log-likelihood is used as a loss function [8]. In each step *k *= 1, ..., *M *the negative gradient of the loss function, evaluated for the current estimate *F*_*k*-1_(*x*; $\hat{\beta }$_*k*-1_) at the observations, is fitted e.g. by standard least squares techniques. The resulting fit *f*_*k*_(*x*; $\hat{\gamma }$_*k*_), which depends on some parameter vector *γ*_*k*_, then is used to updated the overall fit via *F*_*k*_(*x*; $\hat{\beta }$_*k*_) = *F*_*k*-1_(*x*; $\hat{\beta }$_*k*-1_) + *ε*$\hat{f}$_*k*_(*x*; $\hat{\gamma }$_*k*_), where *ε *is some small positive value.

In componentwise boosting a linear predictor of the form *F*_*k*_(*x*; $\hat{\beta }$_*k*_) = *x'*$\hat{\beta }$_*k *_is used and only one element of $\hat{\beta }$_*k *_is updated in each boosting step [9]. The parameter to be updated in step *k *is determined by evaluating fits to the gradient $\hat{f}$_*kj*_(*x*_*i*;_*$\hat{\gamma }$*_*j*_) = $\hat{\gamma }$_*j*_*x*_*ij*_, *j *= 1, ..., *p*, where $\hat{\gamma }$_*j *_is determined by least-squares, and selecting that one that improves the overall fit the most. This results in sparse fits similar to Lasso-like approaches, with many of the estimated coefficients being equal to zero.

For linear models with squared-error loss function, gradient boosting is equivalent to iterative fitting of residuals. This idea has been adapted to the generalized linear model setting as an alternative to the gradient approach [3]. In each boosting step, estimation is performed by a standard Newton-Raphson step, based on a penalized likelihood, where previous boosting steps are incorporated as an offset. An advantage of this offset-based boosting approach is that it allows for very flexible penalty structure, including unpenalized mandatory covariates. Adapting it for survival models would help to resolve the highlighted issues arising when clinical covariates should be included in high-dimensional survival models.

One could also try to adapt existing gradient boosting techniques to allow for unrestricted mandatory components, but we think the offset-based approach is a more natural starting point. Alternatively, approaches such as the grouped Lasso [10,11], which allow for groups of covariates with varying penalization, could potentially be adapted by introducing groups with no penalization. As this has not yet been considered by their authors, and also the group Lasso approach for the Cox model [12] no longer uses simultaneous estimation of all parameters, we do not follow this route here.

In the following will therefore adapt the offset-based boosting approach from [3] for estimating Cox proportional hazards models. The resulting advantage of allowing for unpenalized mandatory components for clinical covariates will be illustrated with data from patients with diffuse large B-cell lymphoma.

## Results and discussion

### Algorithm

The aim of the new *CoxBoost *approach is to estimate the parameter vector *β *for a linear predictor *F*(*x*; *β*) = *x'β *in the Cox proportional hazards model (1). Typical gradient boosting approaches either use all covariates for the fitting of the gradient in each step, e.g. based on regression trees, or, in componentwise boosting, update only one element of the estimate of *β*, corresponding to only one covariate. The flexibility of the offset-based approach in [3] partly is due to considering a flexible set of candidate sets, i.e., a set of sets of covariates, for updating in a specific boosting step. This is adapted for the CoxBoost approach. In boosting step *k *= 1, ..., *M *there are *q*_*k *_predetermined candidates sets of covariates with indices ℐ_*kl *_⊆ {1, ..., *p*}, *l *= 1, ..., *q*_*k*_. For each of these *q*_*k *_sets a simultaneous update of the parameters for the corresponding covariates is evaluated. The candidate set that improves the overall fit the most will then be selected for the update.

With ${\hat{\beta }}_{k-1}=({\hat{\beta }}_{k1},...,{\hat{\beta }}_{kp}{)}^{\prime }$ being the actual estimate of the overall parameter vector *β *after step *k *- 1 of the algorithm, and ${\hat{\eta }}_{i,k-1}={x^{\prime }}_{i}{\hat{\beta }}_{k-1}$ being the corresponding linear predictors, potential updates for the elements of $\hat{\beta }$_*k*-1 _corresponding to ℐ_*kl *_are obtained by maximizing the penalized partial log-likelihood

(3) $$l_{pen}(\gamma_{kl})=\sum_{i=1}^{n}\delta_{i}\left({\hat{\eta }}_{i,k-1}+{x^{\prime }}_{i,{ℐ}_{kl}}\gamma_{kl}-\log\left(\sum_{j=1}^{n}I(t_{j}\leq t_{i})\exp({\hat{\eta }}_{i,k-1}+{x^{\prime }}_{i,{ℐ}_{kl}}\gamma_{kl}\right)\right)-\lambda {\gamma^{\prime }}_{kl}P_{kl}\gamma_{kl}$$

with respect to the parameter vector *γ*_*kl *_of size |ℐ_*kl*_|, where $x_{i,{ℐ}_{kl}}$_*kl *_is the covariate vector for subject *i *containing only those covariates with indices in ℐ_*kl*_. The penalty parameter *λ *which has to be selected, results in a cautious update, if it is large enough. The penalty matrices *P*_*kl *_can be specified separately for each boosting step and each candidate set, which provides considerable flexibility of the CoxBoost approach. Typically these will be diagonal matrices, for penalizing each covariate separately, but by varying the size of the diagonal elements, differential penalization is introduced. In contrast, for gradient boosting approaches the fitting in each step is performed unpenalized and only afterwards the update is multiplied by a small shrinkage factor *ε*, thus applying equal penalization to all covariates. For the present application of the CoxBoost approach we will use only diagonal elements 1 and 0, for "penalization" and "no penalization".

The parameter estimates $\hat{\gamma }$_*kl *_for evaluating the candidate sets are obtained by penalized partial likelihood techniques [13]. Using the starting value ${\hat{\gamma }}_{kl_{0}}=0$, the first Newton-Raphson step is

(4) $${\hat{\gamma }}_{kl}=I_{pen}^{-1}({\hat{\gamma }}_{kl_{0}})U({\hat{\gamma }}_{kl_{0}}),$$

where *U*(*γ*) = (*∂l*/*∂γ*)(*γ*) is the score function and *I*_*pen*_(*γ*) = (*∂*^2^*l*/*∂γ∂γ'*)(*γ*) + *λP*_*kl *_is the information matrix, obtained from the first and second derivatives of the unpenalized partial log-likelihood *l*(*γ*_*kl*_), i.e., (3) without the penalty term. As further updates can take place in later boosting steps, only one Newton-Raphson step is performed.

Given the sets of sets of indices ${ℐ}_{k}=\{{ℐ}_{k1},...,{ℐ}_{q_{k}}\}$, corresponding penalty matrices *P*_*kl*_, *k *= 1, ..., *M*, and the penalty parameter *λ*, the general CoxBoost algorithm is as following:

1. Initialize $\hat{\eta }$_*i*_, _0 _= 0, *i *= 1, ..., *n*, and $\hat{\beta }$_0 _= (0, ..., 0)*'*.

2. Repeat for *k *= 1, ..., *M*

(a) Obtain potential updates $\hat{\gamma }$_*kl *_for the candidate sets ℐ_*kl*_, *l *= 1, ..., *q*_*k*_, via (4).

(b) Determine the best update l* which maximizes the penalized partial log-likelihood (3).

(c) Obtain the updated parameter vector $\hat{\beta }$_*k *_vector via

$${\hat{\beta }}_{kj}=\{\begin{array}{lll} \begin{array}{l} {\hat{\beta }}_{k-1,j}+{\hat{\gamma }}_{kl(j)}\\ {\hat{\beta }}_{k-1,j} \end{array} & \begin{array}{c} j\in {ℐ}_{kl^{*}}\\ j\notin {ℐ}_{kl^{*}} \end{array} & j=1,...,p \end{array},$$

where $\hat{\gamma }$_*kl*(*j*) _is that element of $\hat{\gamma }$_*kl *_that corresponds to $\hat{\beta }$_*k*, *j*_, and update ${\hat{\eta }}_{k,i}={x^{\prime }}_{i}{\hat{\beta }}_{k}$, *i *= 1,..., *n*.

Note that the step size for the updates in part 2c) of the algorithm is 1. This is in contrast to gradient boosting algorithms, where the fits ${\hat{f}}_{k}(x,{\hat{\gamma }}_{k})$ to the gradient are multiplied by some small positive value *ε *before updating. In the CoxBoost algorithm the role of *ε *is taken by the penalty parameter *λ *during estimation. In the following, for unpenalized mandatory components the corresponding elements of the penalty matrix *P*_*kl *_are taken to be zero, resulting in fast building up of coefficient estimates.

#### Componentwise CoxBoost with mandatory covariates

Componentwise CoxBoost, similar to componentwise ridge boosting [3], is obtained when in each boosting step only one element of the overall parameter vector is updated, i.e., ℐ_*k *_= {{1}, ..., {*p*}}, *k *= 1, ..., *M *. In this setup CoxBoost is very similar to the idea of stagewise regression described in [14]. Based on the results given there and in [3] we expected the resulting coefficient paths, i.e., the estimated parameters in the course of the boosting steps, to be very similar to Lasso-like approaches. For strong correlations between covariates, again due to its similarity to stagewise regression, it is expected that the coefficient paths of componentwise CoxBoost are even more stable, i.e., more monotone, than that of Lasso-like approaches [15].

There are two approaches for incorporating mandatory covariates into the CoxBoost algorithm. Given the indices of the mandatory covariates ℐ_*mand*_, the indices from componentwise CoxBoost can be augmented via ℐ_*k *_= {{1} ∪ ℐ_*mand*_, ..., {*p*} ∪ ℐ_*mand*_}, omitting components {*j*} ∪ ℐ_*mand *_where *j ∈ *ℐ_*mand*_. This allows for simultaneous estimation of the parameters of mandatory and optional covariates. When the diagonal elements of the penalty matrices *P*_*kl *_corresponding to ℐ_*mand *_are set to zero, while the others still have a value larger than zero, this furthermore leads to unpenalized estimation of the parameters of the mandatory covariates. When one wants to evaluate whether the optional covariates provide additional predictive power compared to the mandatory covariates, this is the appropriate penalty structure. Alternatively, mandatory covariates can be introduced by updating their parameters before each step of componentwise CoxBoost. This corresponds to ℐ_2*k*-1 _= {ℐ_*mand*_}, ℐ_2*k *_= {{1}, ..., {*p*}} (omitting components {*j*} where *j ∈ *ℐ_*mand*_), *k *= 1, ..., *M*. Again, for evaluating the additional predictive performance obtained from the optional covariates we suggest to use penalty equal to zero for the mandatory covariates.

### Implementation

There are several implementation decisions to be made for the CoxBoost algorithm. At the lowest level, a criterion for selecting the best update l* in each step has to be chosen. Ideally, the penalized partial log-likelihood (3) or some variant of it that incorporates model complexity (such as AIC) would be used. While for a small number of covariates, say *p *< 100, this is computationally unproblematic, for large *p *it is no longer feasible to evaluate this criterion for each candidate set in each step. As an approximation, we therefore propose to employ a penalized version of the score statistic

$$U(\gamma {)}^{\prime }I_{pen}^{-1}(\gamma )U(\gamma )$$

evaluated at ${\hat{\gamma }}_{kl_{0}}$ This is based on a low-order Taylor expansion of the penalized partial log-likelihood (3) and requires no extra computation. In our experiments, selecting boosting step updates by the largest value of this score statistic was very close to selecting by the penalized partial log-likelihood itself, but considerably reduced computation time.

For including mandatory covariates, computational considerations led us to use the CoxBoost variant with separate updating of the mandatory parameters. This avoids frequent inversion of *I*_*pen*_(*γ*), because in the componentwise updating step of this variant for the optional covariates this reduces to a simple division. The CoxBoost algorithm has two tuning parameters, the penalty parameter *λ *and number of boosting steps *M*. While selection of the latter is critical to avoid overfitting, the penalty parameter is of minor importance, as long as it is large enough. We therefore suggest to select only the number of boosting steps by a procedure such as cross-validation. The penalty parameter *λ *is selected only very coarsely such that the corresponding selected number of boosting steps *M *is larger than 50. This approach was seen to work well for offset-based boosting for generalized linear models [3].

The algorithm has been implemented in the statistical environment R [16] in the package "CoxBoost", which is available from the authors.

### Example

We illustrate the CoxBoost algorithm with the diffuse large B-cell lymphoma data from the study in [17]. A review of attempts to build predictive survival models from such data is found in [18]. There is a potentially censored survival time response for 240 patients with a median follow up of 2.8 years, where 57% of the patients died during that time. For prediction there are 7399 microarray features available. In addition, the International Prognostic Index (IPI), a well-established prognostic score derived from five clinical covariates [19], is available for 222 patients. As we want to investigate whether the microarray features increase predictive performance compared to a purely clinical model based on the IPI, analyses are restricted to this smaller set of patients. Missing values for the microarray features were imputed as described in [20].

In [17] the data is split into a training set where the parameters are estimated, and a test set where prediction performance is evaluated. The disadvantage of this is that not all data is available for model building and parameter estimation. We employ an alternative approach [20], based on bootstrap samples, which allows to use all observations for model fitting, but nevertheless results in accurate prediction error estimates. For evaluation of prediction performance the Brier score is used, i.e., the (expected) squared difference between predicted survival probability at a time *t *and the true state (1 for being still under risk, and 0 if an event occurred). This can be be plotted as a function of time, resulting in prediction error curves. For estimation of the latter, prediction error estimates obtained from single bootstrap samples are aggregated into a .632+ estimate. An additional summary measure is obtained when for every single bootstrap sample a .632+ prediction error curve is calculated and integrated (in our case up to time 10). See the Methods section for more details.

As a conservative reference for performance comparison the Kaplan-Meier prediction is used, a non-parametric estimate of the survival probability over time. That way it can be checked whether procedures potentially perform worse than a prediction that does not use any covariate information at all. The performance of componentwise CoxBoost is furthermore compared to that of gradient boosting for the Cox model [1] (R package "mboost" [21]) and that of CoxPath, a Lasso-like path algorithm for fitting the Cox model [2] (R package "glmpath" [22]). For fitting models with these procedures only the microarray features (i.e., not the IPI) are used. In addition, componentwise CoxBoost with the IPI as an additional optional and as an unpenalized mandatory covariate is compared to a simple Cox model that has the IPI as its only covariate. The tuning parameters, i.e., the number of boosting steps and the number of path algorithm steps, are chosen by 5-fold cross-validation with respect to the partial log-likelihood. All other settings are at the default values of the respective implementations.

**Figure 1:**
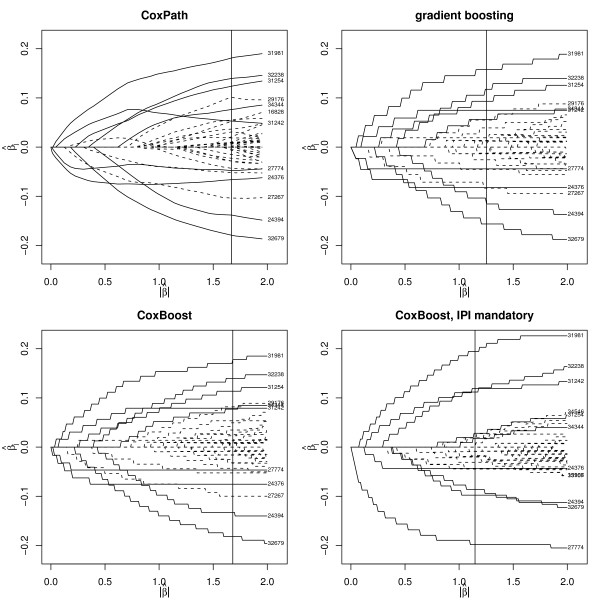
**Coefficient paths for CoxBoost**. Estimated parameters plotted against the norm of the parameter vector for CoxPath (top left), gradient boosting (top right), componentwise CoxBoost (bottom left), and CoxBoost with a mandatory covariate (bottom right). CoxPath steps and boosting steps selected by cross-validation are indicated by vertical lines. Covariates selected by all approaches up to this number of steps are indicated by solid curves, the others by dashed curves. For them and other strong covariates the UNIQID is given.

Before looking at prediction performance, we investigate the influence of unpenalized mandatory covariates on the coefficient paths, i.e., the parameter estimates for the individual covariates plotted against the norm of the parameter vector (which increases in the course of the CoxPath/boosting steps). Figure 1 shows the coefficient paths for CoxPath, gradient boosting, componentwise CoxBoost, and componentwise CoxBoost with the IPI as a mandatory covariate (where the parameter estimates for the IPI are not shown). The estimates corresponding to the number of CoxPath steps and the number of boosting steps selected by cross-validation are indicated by vertical lines. Covariates that receive non-zero parameter estimates by all four approaches in that cross-validation solutions are indicated by solid curves, the others by dashed curves. For the former, and other microarray features with corresponding parameter estimates that are large in absolute value, the UNIQIDs are given in the right margins of the plots.

It is seen that the coefficients paths for componentwise CoxBoost, gradient boosting and CoxPath are very similar. For the latter they are a bit more unstable, in the sense that they are not monotone, which is to be expected based on the results in [15]. Nevertheless, the six microarray features with the largest absolute value of the parameter estimates are the same for all three approaches.

The coefficient paths of CoxBoost with the IPI as a mandatory covariate are different, with only a small number of distinct covariates receiving large parameter estimates. The reason for this might be that the mandatory covariate already explains much of the variation in the response and there is less incentive to boost a large number of parameters to fit the remaining variability. The number of boosting steps selected by cross-validation (indicated by vertical lines) also supports this, as it is smaller compared to simple componentwise CoxBoost when IPI is present as a mandatory covariate. In this example, including an unpenalized mandatory covariate also changes the ranking of the microarray features with respect to the absolute values of the parameter estimates. After inclusion of the IPI the microarray feature with UNIQID 27774 is associated with a strong protective effect, while it seemed to be of minor importance judged by the other fits. In contrast, the feature with UNIQID 32679 is deemed to be less important when the IPI is included as an unpenalized mandatory covariate. So the latter clearly changes the interpretation of the fitted models.

**Figure 2:**
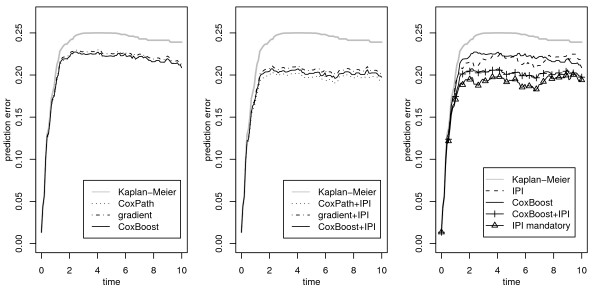
**.632+ prediction error estimates**. .632+ prediction error curve estimates for microarray-only models (left panel) fitted by CoxPath, gradient boosting, and componentwise CoxBoost, for models including the IPI as an additional optional covariate (middle panel), and for the CoxBoost fit that incorporates the IPI as a mandatory covariate (right panel). The Kaplan-Meier benchmark is indicated by gray curves.

The left panel of Figure 2 shows the .632+ prediction error estimates for all models that incorporate only microarray features, i.e., CoxPath (dotted curve), gradient boosting (dash-dotted curve), and componentwise CoxBoost (solid curve). It is seen that all three perform very similar. The prediction error is well below the Kaplan-Meier benchmark (gray curve), which does not employ any covariate information. This is not self-evident, as for example in the evaluation in [20] some other procedures failed with respect to this criterion. So the offset-based boosting approach does not seem to result in a loss of prediction performance and it therefore is a reasonable basis for an approach incorporating unpenalized mandatory covariates. While according to the prediction error curve estimates there seems to be no disadvantage for CoxPath, the out-of-bag partial log-likelihood, i.e., the mean partial log-likelihood evaluated for the observations not in the respective bootstrap samples, is the smallest for this procedure (-183.8). For gradient boosting and componentwise CoxBoost it is -181.5 (with standard errors of about 1.4), i.e., also with respect to this error measure there seems to be no disadvantage of using the CoxBoost approach. A similar pattern is seen for models that incorporate the IPI as an optional covariate in addition to microarray features (middle panel of Figure 2). There is a general improvement over models that did not include the IPI, with all procedures again performing very similar. According to the prediction error curve estimates there may be a slight advantage for CoxPath, which seems to gain the most prediction performance. However, the out-of-bag partial log-likelihood is again the smallest for this procedure (-180.3), while for gradient boosting it is -180.0, and for CoxBoost it is even -177.8.

The effect of various ways for dealing with clinical covariates is illustrated in the right panel of Figure 2. There the .632+ prediction error estimate for componentwise CoxBoost is given, together with prediction error curves for CoxBoost approaches that incorporate the IPI, either as an optional (curve with plus symbols) or as a mandatory covariate (curve with triangles). In addition, the estimated prediction error curve for a standard Cox model that incorporates only the IPI is given (dashed curve). The performance of a microarray-only CoxBoost fit (solid curve) is roughly similar to the Cox model that includes only the clinical information from the IPI (out-of-bag partial log-likelihood: -177.8), with an advantage for the latter for early prediction times. So both types of model might contain the same amount of information. The question whether the microarray features contain information that is different from that of clinical covariates is therefore still unanswered. When, as a first step, the IPI is included as an additional optional covariate for componentwise CoxBoost, as already noted, there is a distinct increase in prediction performance compared to componentwise CoxBoost based only on microarray features. So the two types of covariates seem to contain (at least partially) different information. When the IPI is included as an unpenalized mandatory covariate the performance increases even more (out-of-bag partial log-likelihood: -175.3). This shows that it is really necessary to assign the IPI this special role, as otherwise it cannot exert its full predictive potential. Here the flexibility of the CoxBoost approach allows to incorporate subject matter knowledge, i.e., knowing that the IPI is a good predictor, to increase predictive performance. CoxBoost with the IPI as a mandatory covariate also allows for a valid comparison to the Cox model that contains IPI as its only covariate. As in both models the parameters for the IPI are estimated unpenalized, the exact additional value of the microarray features in terms of predictive performance can be seen from the difference between the two curves.

**Figure 3:**
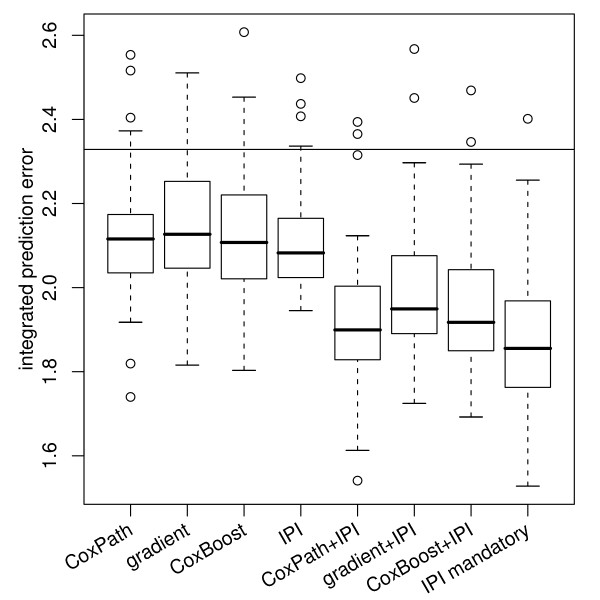
**Variability of the .632+ prediction error estimates**. Integrated prediction error curve estimates from single bootstrap samples for CoxPath, gradient boosting, componentwise CoxBoost, and an IPI-only Cox model ("IPI"), for corresponding models where the IPI is included as a an additional optional covariate ("...+IPI"), and for CoxBoost fits that incorporate the IPI as a mandatory covariate ("IPI mandatory"). The Kaplan-Meier benchmark value is indicate by a horizontal line.

Figure 3 shows boxplots of the integrated prediction error estimates (up to time 10) calculated for the single bootstrap samples, to convey an impression of the variability underlying the estimates in Figure 2. It is seen that the conclusions drawn from the prediction error curve estimates hold, even when variability is taken into account. For microarray-only models and models that incorporate the IPI only as an optional covariate, CoxPath, gradient boosting, and CoxBoost perform very similar, but when the IPI is incorporated as an unpenalized mandatory covariate, there is an advantage in terms of prediction performance for CoxBoost.

## Conclusion

Modern techniques for the fitting of predictive survival models, such as Lasso-like approaches and boosting, are capable of handling the large number of covariates often arising in bioinformatics applications, e.g. from microarrays. What has been missing is an approach for incorporating mandatory covariates into such models. We therefore adapted an offset-based boosting approach, which allows for flexible penalization of covariates, for the estimation of Cox proportional hazard models.

The flexible penalty structure of the new approach allows for unrestricted estimation of the parameters for mandatory covariates. As seen in an example application, this also influences the coefficient paths for the optional covariates, in this case resulting in a more transparent structure. The main benefit, on the one hand, was increased prediction performance by combining clinical and microarray information. On the other hand, the increase of prediction performance over a microarray-only model and a purely clinical predictive model helped to answer the question about the additional benefit arising from microarray technology for predicting survival. In the example, including a mandatory covariate also affected the ranking of microarray features with respect to absolute value of the parameter estimates and therefore potentially changed the clinical implications of the result.

Componentwise gradient boosting approaches could potentially also be adapted for incorporating unpenalized mandatory covariates. However, simply augmenting the componentwise base learners by mandatory components would not be sufficient, as in gradient boosting the base learner fits are multiplied by some small constant *ε *before adding them to the overall fit. Therefore the building up of the coefficient estimates for mandatory covariates would still be rather slow. Introducing intermediate steps with *ε *= 1, where only mandatory covariates are updated, could address this. However, the offset-based boosting approach, which we used as a basis for the CoxBoost algorithm, more naturally allows for unpenalized mandatory components.

Incorporating unpenalized mandatory covariates is only one of the many possible ways of leveraging clinical information and subject matter knowledge using the proposed boosting approach. For example, information from clustering of the microarray features could be incorporated, by distributing boosting steps over a set of clusters. Further refinements of the boosting scheme and the penalization structure could be devised, for further increasing prediction performance and to more generally increase the usefulness of the resulting predictive model.

## Methods

### Measures of prediction error

By estimating the parameter vector $\hat{\beta }$ of a Cox model (1), a risk prediction model

$$\hat{r}(t|x_{i})=\exp(-{\hat{\Lambda }}_{0}(t)\exp({x^{\prime }}_{i}\hat{\beta }))$$

is obtained, where $\hat{\Lambda }$_0_(*t*) denotes the Breslow estimator of the cumulative baseline hazard $\Lambda_{0}(t)=\int_{0}^{t}\lambda_{0}(s)ds$. It predicts the event status

*Y*_*i*_(*t*) = *I*(*T*_*i *_> *t*),

where *I*() takes the value 1 if its argument is true and 0 otherwise. *T*_*i *_is the survival time of subject *i*, that is unobserved in case of censoring. The observed time *t*_*i *_therefore is *t*_*i *_= *min*(*T*_*i*_*, C*_*i*_), where *C*_*i *_is the censoring time.

The *true prediction error curve *then is

$$Err(t;\hat{r})=E[{\left(Y(t)-\hat{r}(t|x)\right)}^{2}].$$

It can be estimated from a sample via

(5) $$\bar{err}(t;\hat{r})=\frac{1}{n}\sum_{i=1}^{n}{\left(Y_{i}(t)-\hat{r}(t|x_{i})\right)}^{2}W(t;\hat{G}),$$

where weights *W*(*t*; $\hat{G}$) have to be introduced to account for censoring. To obtain a consistent estimate of the true prediction error curve they have to be chosen to be

$$W(t;\hat{G})=\frac{I(t_{i}\leq t)\delta_{i}}{\hat{G}(t_{i}-|x_{i})}+\frac{I(t_{i}>t)}{\hat{G}(t|x_{i})},$$

where $\hat{G}$(*t|x*) is a consistent estimate of *P*(*C *> *t*|*x*). We use a Kaplan-Meier estimator for the latter. For more details see [23].

### .632+ prediction error estimates

Evaluating (5) with the data that was used for estimating $\hat{\beta }$ potentially underestimates the prediction error. We therefore generate sets of indices J_*b *_⊂ {1, ..., *n*}, *b *= 1, ..., *B*, for *B *= 100 bootstrap samples, each of size 0.632*n*. Sampling without replacement is used to avoid a potential complexity selection bias (i.e., for selecting the number of boosting steps or CoxPath steps) indicated e.g. in [24]. The bootstrap cross-validation error estimate is then obtained by

(6) $${\hat{Err}}_{B0}(t,\hat{r})=\frac{1}{B}\sum_{b=1}^{B}\frac{1}{b_{0}}\sum_{i\notin J_{b}}{\left(Y_{i}(t)-{\hat{r}}_{b}(t|x_{i})\right)}^{2}W(t,\hat{G}),$$

where *b*_0 _is the number of observations not in J_*b*_, i.e., 0.368*n*, and $\hat{r}$_*b *_is the model fitted to the observations with indices in J_*b*_.

As (6) is known to be biased upwards, we use the .632+ estimate

(7) $${\hat{Err}}_{.632+}(t,\hat{r})=\{1-\omega (t)\}\bar{err}(t,\hat{r})|\omega (t){\hat{Err}}_{B0}(t,\hat{r}),$$

with $\hat{\omega }$(*t*) = .632/(1 - .368 $\hat{R}$(*t*)), where $\hat{R}$(*t*) is the relative overfitting rate $\hat{R}(t)=\frac{{\hat{Err}}_{B0}(t,\hat{r})-\bar{err}(t,\hat{r})}{NoInf(t,\hat{r})-\bar{err}(t,\hat{r})}$, with $NoInf(t,\hat{r})=\frac{1}{n^{2}}\sum_{j=1}^{n}\sum_{i=1}^{n}{\left\{Y_{i}(t)-\hat{r}(t|x_{j})\right\}}^{2}W(t,\hat{G})$. For more details see [25].

As a summary measure we propose to use the integrated prediction error estimate

(8) $$I{\hat{Err}}_{.632+}(t^{*},\hat{r})=\int_{0}^{t^{*}}{\hat{Err}}_{.632+}(s,\hat{r})ds.$$

For getting and impression of the variability underlying (7) and (8), (7) is calculated separately for every bootstrap sample, i.e., the outer sum in (6) reduces to one term, and the corresponding integrated prediction error estimates are obtained. The variability of the resulting *B *= 100 individual integrated prediction error estimates can then be compared between different prediction models, e.g. by boxplots.

## Authors' contributions

HB developed and implemented the initial version of the proposed algorithm, applied it to the example data and wrote most of the manuscript. MS contributed design decisions for the algorithm, helped with interpretation of the results for the example data and revised the manuscript.
